# Supplementary material for: Delayed repolarization and ventricular tachycardia in patients with heart failure and preserved ejection fraction
Source: PLoS One. 2021 Jul 13;16(7):e0254641. doi: 10.1371/journal.pone.0254641 (PMC8277017; doi:10.1371/journal.pone.0254641)
Supplement: Attachment — Submitted filename: Response to Reviewers.docx [file pone.0254641.s001.docx]

**Response to Reviewers**

Reviewer #1: The authors of “Delayed repolarization and ventricular tachycardia in patients with Heart Failure Preserve Ejection fraction” attempts to explore the association between HFpEF and ventricular arrhythmias. The hypothesis presented is relevant and statistically methods appear sound. Caution, however, needs to be made in some of the conclusions drawn from this rather small retrospective dataset, among some other minor comments.

We appreciate the reviewer’s favorable comments. We have revised our manuscript following your comments. Please see the manuscript with tracked changes with line numbers.

1. Caution concluding the enhance propensity of VT contributes to sudden death in these patients as there was no long term follow up or outcome data presented. As mentioned by the authors “non-sustained VT has been recorded in the variety of patients, from healthy individual to patient with significant heart disease. The actionability of non-sustained VT is high debateable …..” I would encourage the authors to focus on the increase prevalence of VT demonstrated by the data presented, underlying mechanisms and be cautious in suggesting a direct association with NSVT and SCD.

We agree with the reviewer’s point. Hence, we removed our conjecture that increased propensity to VT contributes to sudden death.

Line 165-166

Deleted “consistent with the conjecture that an enhanced propensity to VT contributes to sudden death in these patients.”

Line 180

Changed “sudden death” to “non-sustained VT”.

2. In the statistical methods the authors have stated that results were considered significant at p < 0.05, please avoid using qualifying words through the manuscript such as “modestly” or “strong trend” to imply statistical significance.

We have modified our manuscript as below.

Line 177-179

Before

“Here we have described, in human patients, associations among HFpEF, VT prevalence, QT interval prolongation and, modestly, QRS prolongation.”

After

“Here we have described, in human patients, associations among HFpEF, VT prevalence, and QTc interval prolongation.”

Line 133-134

Before

“QRS width showed a strong trend toward widening in patients with HFpEF (p=0.051).”

After

“QRS width was not different between control and HFpEF patients.”

3. In the methods section please clarify if the patients included were inpatients, ambulatory care patients or both.

We included inpatients and outpatients. We modified the methods section as described below:

Line 73-75

“We identified all patients (inpatients and outpatients) who underwent ambulatory monitoring of ECG by Zio Patch (iRhythm, San Francisco, CA) for any reason from January 2016 to December 2018 (3 years, N=2,913, Figure 1).”

4. Considering echocardiographic data was used to define HFpEF, these data should be provided.

We have added echocardiographic data (MV inflow E wave, A wave, E/A ratio, lateral e’ wave, E/e’ ratio, TR velocity and LA volume index) to Table 1.

5. Were laboratory values available around the time of echocardiography, specifically NT-proBNP or metabolic panel?

We have added BNP and metabolic panel (Na, K, Ca and Cr) to Table 1.

6. If these data suggest an enhance propensity to VT in HFpEF, why does the frequency of arrhythmias, specifically VT, not increase with severity of diastolic dysfunction.

This is a great point. Although we expect positive relationship between VT and diastolic dysfunction, due to low number of patients, it was not significant.

7. If patients in the HFpEF were taking more rate controlling medication, is there an explanation for why was there no difference in heart rates between to two groups?

This is also a good point. Although more patients were taking beta-blockers in the HFpEF group, HR on ECG were not significantly different. HF patients usually have increased baseline HR (partially due to increased sympathetic tone) and this might explain increased baseline HR values. Due to increased baseline HR, beta-blockers were only able to reduce HR of HFpEF patients to the level of control patients. This is only our speculation, but it is hard to prove in our cohort and due to the nature of retrospective medical record review.

8. While the initial pool of patients is large, the actually dataset used for analysis is small and this should be specifically mentioned in the limitations.

We have included this limitation in our discussion:

Line 212-214

“Third, although the initial pool of patients is large, the actual dataset used for analysis is small. This is mainly due to the limited use of Zio Patch monitoring.”

Reviewer #2: This study examined Zio Patch data in patients with a clinical diagnosis of HFpEF. Patients with normal diastolic and systolic function served as controls. The results showed that VT was more prevalent in patients with HFpEF (versus those without this diagnosis).

We appreciate the reviewer’s favorable comments. We have revised our manuscript following your comments. Please see the manuscript with tracked changes with line numbers.


Comments:

-How much time was there between the Zio Patch and TTE?

For most of the patients (87/97 control and 100/110 HFpEF), it was within a week. Only for 2/97 control patients and 2/110 HFpEF patients, Zio Patch was performed after 30 days.

This was included in the manuscript.

Line 120-121

“Most of the patients underwent Zio Path within a week from echocardiogram (only 2 patients in each group underwent Zio Patch after 30 days of echocardiogram).”

-The authors used the ASE guidelines to adjudicate evidence of diastolic dysfunction however there is significant controversy on how age influences diastolic parameters – namely septal and lateral e’ velocities. An older patient with lower e’ velocities may also have higher E/e’ ratios. These patients might be categorized with diastolic dysfunction when this may not be the case.

We absolutely agree with the reviewer’s point. Older patients have higher E/e’ ratio due to low e’ wave. But we have extensively reviewed medical records and identified HFpEF patients with diastolic dysfunction and HF symptoms. We have excluded patients with diastolic dysfunction without HF symptoms.

We have modified our methods section as below.

Line 89-90

“Patients with diastolic dysfunction (due to old age) without documented HF symptoms were excluded”.

-How were the medical records used to determine the presence of clinical HFpEF? Was BNP used? What criteria were used to classify patients as HFpEF. Relying on the coded medical chart alone leaves a lot of opportunity to introduce errors.

We have extensively reviewed medical records and included patients with HF symptoms and diastolic dysfunction on echocardiogram. We did not rely on the code because, as the reviewer pointed, it can falsely tag patients with diastolic dysfunction as HFpEF. We also included data of BNP in the table 1. BNP is a good marker of HF but some of the HFpEF have normal BNP level. We used BNP to confirm the diagnosis of HFpEF but did not solely rely on BNP level.

-Rather than say mild, moderate, severe diastolic dysfunction, use grade I, II, or III if this is what the authors are suggesting. I am also surprised that no patients had grade III diastolic dysfunction using criteria E/A>2.

We have changed our manuscript from mild, moderate and severe to grade I, II and III diastolic dysfunction as well as our tables 1 and 3. We are also surprised that we didn’t have any grade III diastolic dysfunction in our patients.

-It’s odd that patients with other risk factors did not have higher chance of VT (namely CAD). This might be due to a sample size issue. Could the authors expand the date range if Zio Patch data was available before 2016.

Although it is desirable to expand our study to before 2016 but this study was specifically designed for patients from January 2016 to December 2018. Such a study will need separate IRB protocol and additional research staff. With the appropriate funding, we would like to perform a prospective study to investigate the associations of QTc prolongation, VT and sudden death. Hopefully this large-scale prospective study will address that important question.

-I’m sure a number of these patients underwent cardiac MRI…of the ones who underwent CMR, what fraction had myocardial LGE present in this study and how does this impact the data.

We have looked at how many patients underwent MRI in our study and only 5/97 control patients and 2/110 HFpEF patients underwent MRI. All of 5 control patients did not have LGE and only 1 of 2 HFpEF patient showed small focal fibrosis in inferior wall. Hence, we are not able to comment on the impact of LGE on VT in patients with HFpEF.

-The wording “mode of exodus” in the abstract , introduction, and discusssion is odd…perhaps change to “most common cause of mortality”

We have changed exodus to mortality throughout our manuscript.
